# Integrated Analysis of TME and Hypoxia Identifies a Classifier to Predict Prognosis and Therapeutic Biomarkers in Soft Tissue Sarcomas

**DOI:** 10.3390/cancers14225675

**Published:** 2022-11-18

**Authors:** Ruiling Xu, Lin Qi, Xiaolei Ren, Wenchao Zhang, Chenbei Li, Zhongyue Liu, Chao Tu, Zhihong Li

**Affiliations:** 1 Department of Orthopedics, The Second Xiangya Hospital, Central South University, Changsha 410010, China; 2 Hunan Key Laboratory of Tumor Models and Individualized Medicine, The Second Xiangya Hospital, Changsha 410010, China

**Keywords:** soft tissue sarcomas, hypoxia, tumor microenvironment, prognosis, therapy strategizing

## Abstract

**Simple Summary:**

Soft tissue sarcomas are uncommon and diverse solid tumors with high risks that have a poor prognosis. Tumor microenvironment (TME) and hypoxia play critical roles in tumor development. Therefore, we aimed to determine whether linking hypoxia-related parameters to TME cells could improve prognosis and treatment outcomes. The Hypoxia-TME classifier was first proposed by us using TCGA-SARC court (*n* = 258) and fusion data from GSE63157 and GSE30929 (*n* = 225). This classifier is capable of correctly classifying patients based on their prognosis and immune type. In addition, immunotherapy and chemotherapy programs were provided in a more specific manner. Several key genes were identified for future research as a result of the classification results.

**Abstract:**

Soft tissue sarcoma (STS) is one of the rarest but most aggressive cancer. It is important to note that intratumoral hypoxia and tumor microenvironment (TME) infiltration play a significant role in the growth and therapeutic resistance of STS. The goal of this study was therefore to determine whether linking hypoxia-related parameters to TME cells could provide a more accurate prediction of prognosis and therapeutic response. An analysis of 109 hypoxia-related genes and 64 TME cells was conducted in STS. Hypoxia-TME classifier was constructed based on 6 hypoxia prognostic genes and 8 TME cells. As a result, we evaluated the prognosis, tumor, and immune characteristics, as well as the effectiveness of therapies in Hypoxia-TME-defined subgroups. The Lowplus group showed a better prognosis and therapeutic response than any other subgroup. It is possible to unravel these differences based on immune-related molecules and somatic mutations in tumors. Further validation of Hypoxia-TME was done in an additional cohort of 225 STS patients. Additionally, we identified five key genes through differential analysis and RT-qPCR, namely, ACSM5, WNT7B, CA9, MMP13, and RAC3, which could be targeted for therapy. As a whole, the Hypoxia-TME classifier demonstrated a pretreatment predictive value for prognosis and therapeutic outcome, providing new approaches to therapy strategizing for patients.

## 1. Introduction

Soft tissue sarcomas (STS) are a diverse group of rare and highly aggressive solid tumors that originate from mesenchymal tissue [1,2]. Approximately 1% to 2% of all new adult cancers are caused by these diseases [3,4,5]. There is a 12- to 16-month survival rate for patients with metastatic disease, and the two-year survival rate is approximately 30%. The outcome for these patients has been poor despite the development of several novel therapies or combinations of chemotherapy [3,6]. Consequently, STS remains a medical need that needs to be addressed.

It is important for STS that the tumor microenvironment (TME) is critical, including the population of immune cells as well as nonimmune cells [7]. Considering that the TME contains a variety of cells essential for antitumor immunity, studying cell composition could provide not only prognostic information but also insight into immunotherapy efficacy [7,8,9]. Consequently, it is crucial to gain a better understanding of how TME composition relates to tumor aggressiveness and therapy response. There are also many non-immune cells within the TME, including stromal cells, that interact with cancer cells [7]. Therefore, we proposed that a more global cellular landscape approach might be a more effective way to predict prognosis and therapy.

As a general feature of TME, hypoxia can contribute to metastatic spread [10,11,12,13]. There is also an association between hypoxia and distant relapse in localized STS [14]. Animal sarcoma models with hypoxic cell populations exhibited reduced lung metastasis after adjuvant administration of systemic agents [15,16,17]. In addition to influencing tumor cells, hypoxia triggers a variety of events in TME and affects many TME surrounding cells as well, which play a crucial role in tumorigenesis, promoting tumor aggression and inhibiting antitumor responses [18,19,20], such as hypoxic cells that tend to switch to glycolytic metabolism, resulting in higher lactate levels, which aggravates the acidification of immunosuppressive TME [21]. Additionally, hypoxic microenvironments enhance immunosuppressive cell infiltration (e.g., M2 macrophages, myeloid-derived suppressor cells, regulatory T cells) [22,23,24,25]. The TME experiences high levels of hypoxia, which inhibits T cell-mediated immunity strongly, and causes immune escape [26,27]. In addition, tumor tissues lack adequate levels of intracellular H_2_O_2_ that hinder chemodynamic treatment [28]. Researchers have shown that hypoxia-reactive drug delivery nanosets can promote cancer chemoimmunotherapy by overcoming tumor immune tolerance induced by hypoxia [29]. In brief, a strong intratumoral Hypoxia-TME cell cross-interaction exists [30,31,32].

As far as we know, no study has reported combining hypoxia and a comprehensive cellular landscape to examine the STS tumor microenvironment. A Hypoxia-TME signature may improve clinical classification as well as therapeutic outcomes for patients with STS considering the effects of hypoxia modifiers [33] and immunotherapy [34]. As a result, we developed a Hypoxia-TME classifier for predicting prognosis and therapeutic response by incorporating TME cells and hypoxia genes. Furthermore, we identified key differential genes to indicate the direction of study into the TME/hypoxia relationship.

## 2. Materials and Methods

### 2.1. Date Source

Downloading of RNA sequencing profiles (TCGA TARGET GTEx cohort) was done using the UCSC browser (http://xenabrowser.net/, accessed on 5 May 2022, Table S1). A download of the clinical data from the TCGA-SARC cohort was conducted through cBioPortal (http://www.cbioportal.org/, accessed on 21 October 2021, Table S2). Based on the combination of expression data and clinical data, 258 STSs patients were identified with complete information. The TCGA-SARC cohort consisted of 104 patients with leiomyosarcoma (LMS), 59 patients with dedifferentiated liposarcoma (DDLPS), 49 patients with undifferentiated pleomorphic sarcoma (UPS), 25 patients with myxofibrosarcoma (MFS), and 21 patients with other STS. We extracted RNA sequencing maps of 395 normal soft tissues (adipose viscera (omentum), Table S3) in order to compare TCGA-SARC with the GTEX cohort and to determine genes with aberrant expression in tumors. Using log2 (TPM + 1), the input TPM was transformed, and added 1 for each TPM value, to avoid divergence. Moreover, we downloaded the gene expression profiling and clinical data of Ewing sarcoma (GSE63157) and liposarcoma (GSE30929) to form the independent validation cohorts. We created an independent validation cohort consisting of 225 patients by combining gene expression profiles (Table S4) and clinical data from GSE63157 and GSE30929 (Table S5). A single-cell dataset of synovial sarcoma (GSM3770931) was collected and analyzed (Table S6).

### 2.2. Identification of Hypoxia Genes and TME Cells Associated with Prognosis

From the KEGG pathway “hsa04066”, 109 hypoxia-related genes were identified within the HIF-1 signaling pathway (Table S7) [35]. In order to analyze the tumor immune microenvironment using transcriptomic data [36], the xCell score was calculated using the xCell algorithm. On the basis of transcriptomes of all tumor samples collected, this method calculates 64 types of TME cells, which outperforms all other approaches (Table S8) [37]. Univariate Cox regression analyses were performed on hypoxia-related genes and TME cells to determine their prognosis. The final results of the analysis indicated that 6 hypoxia-related genes and 8 TME cells were statistically significant predictors of prognosis in STSs. Using the “glmnet” R package (Version 4.1-2), they were further entered into LASSO (least absolute shrinkage and selection operator) Cox regression analysis to narrow down the scope of gene selection. In addition, the risk score was calculated using the formula below:

(1) $$Hypoxia\;score=\sum_{i}^{6}X_{i}*Y_{i}\;(X_{i}:coefficients\;of\;the\;gene\;i,Y_{i}:expression\;values\;of\;the\;gene\;i)$$

(2) $$TME\;score=\sum_{i}^{8}X_{i}*Y_{i}\;(X_{i}:coefficients\;of\;the\;TME\;cell\;i,Y_{i}:expression\;values\;of\;the\;TME\;cell\;i)$$

### 2.3. Establishment of Hypoxia-TME Prognostic Model

According to the hypoxia classifier, TCGA-SARC cohorts were divided into high-risk and low-risk groups. Similarly, based on the TME cell classification, TCGA-SARC cohorts were also divided into high and low-risk groups. KM plots were used to demonstrate the survival differences between high-risk and low-risk groups in the TCGA-SARC cohort. A second external validation was undertaken using GSE63157 and GSE30929, which was divided into similar groups based on the same critical point.

After that, *hypoxia* and *TME scores* were combined to create the *Hypoxia-TME* classifier. Subgroups of tumors were then identified: Highplus (high *hypoxia score*/high *TME score*), Middle (high *hypoxia score*/low *TME score* and low *hypoxia score*/high *TME score*) and Lowplus (low *hypoxia score*/low *TME score*). The score was calculated using the formula below:

(3) $$Hypoxia/TME\;Score=\frac{\left(Hypoxia\;score+TME\;score\;\right)}{2}$$

Evaluation of prediction accuracy was conducted using the “timeROC” R package (version 0.4). In addition, the COX regression analysis was performed on a multivariate means of clinical characteristics, including age, gender, metastasis, and histological type, to analyze the Hypoxia-TME risk model. An illustration of the predictive model was presented by nomograms, followed by a calibration curve evaluation.

### 2.4. DEGs Analysis, Gene Set Enrichment Analysis, and Tumor SOMATIC mutation

Using the “limma” package in R, differentially expressed genes (DEGs) were analyzed. With the help of the clusterProfiler R package (Version 4.0.4), we performed an enrichment analysis using Gene Ontology (GO) and Kyoto Encyclopedia of Genes and Genomes (KEGG). Using the “GSVA” (Version 1.40.1) and “gsease” (Version 1.54.0) R packages, 16 immune cell infiltrations and 13 related functions were revealed. By using previously described methods, we calculated each tumor’s tumor mutational burden (TMB) score [38].

### 2.5. Single-Cell Analysis and Chemotherapeutic Response Prediction

To cluster single cells, the Seurat R package version 1.4.0.1 was used (https://github.com/satijalab/seurat, accessed on 5 May 2022). AUCell was used to calculate hypoxia and immune pathway AUC scores in each cell [39].

The chemotherapeutic response for each group according to the largest publicly available pharmacogenomics database [the Genomics of Drug Sensitivity in Cancer (GDSC), https://www.cancerrxgene.org/, accessed on 25 October 2021]. In order to estimate half of the maximum inhibitory concentration (IC50), the prediction process was implemented using the R package “pRRophetic”. By using the GDSC training set, tenfold cross-validation was conducted to determine the prediction accuracy [40,41].

### 2.6. Cell Culture and Cell Lines

Obtaining the human skin fibroblast cell line (HSF) and its culture media was accomplished by Fenghui Biotechnology Company of China (Hunan). It was provided by the American Type Culture Collection (ATCC) that the human synovial sarcoma cell line (SW-982) be used. We obtained cells from Procell Life Science & Technology Co., Ltd. (Wuhan, China) that are derived from human liposarcoma (SW872). Cell lines for human synovial sarcomas (hSS-005R) were created in Hunan Key Laboratory of Tumor Model and Individualized Medicine. SYO-1 is a cell line provided by Massachusetts General Hospital, Department of Surgical Oncology. Dulbecco’s modified Eagle medium (DMEM) (Gibco, NY, USA) was used to culture SW-982, SW-872, hSS-005R, and SYO-1. 1% penicillin-streptomycin (NCM Biotech. Suzhou, China) and fetal bovine serum (Gibco, NY, USA) were added to the cell culture medium. Humidified incubators (Thermo Fisher Scientific. Waltham, MA, USA) were used to maintain cells at 37 °C and 5% CO_2_.

### 2.7. Quantitative Real-Time PCR

Isolation of total cellular RNA was performed with RNA Express Total RNA Kit (M050, NCM Biotech. Suzhou, China) [42]. RevertAid First Strand cDNA Synthesis Kit (Thermo. Waltham, MA, USA) was used to reverse-transcribe the RNA. RT-qPCR was performed on the StepOne Plus (Applied Biosystems. Waltham, MA, USA) by utilizing SYBR Green qPCR Master Mix (2×) (Bimake. Houston, TX, USA). RT-qPCR was conducted using lyceraldehyde-3-phosphate dehydrogenase (GAPDH) as an internal control gene. A list of primers was given in Table 1.

### 2.8. Statistical Analysis

The data analysis was carried out using R (Version 4.2.1). To determine whether there is a difference in gene expression between the two groups, Wilcoxon rank sum tests were performed, and *p* values were determined for each gene. To analyze survival, the log-rank test was performed along with the KM curve. Spearman’s correlation analysis was conducted to examine gene expression in correlation with survival. Using Fisher’s exact test, we compared clinical characteristics of two groups. With COX regression analysis, multivariable factors were evaluated and hazard ratios are calculated with a 95% confidence interval (CI). There were three statistically significant differences: * *p* < 0.05, ** *p* < 0.01, *** *p* < 0.001, **** *p* < 0.0001.

## 3. Results

### 3.1. Identify Hypoxia and TME Differences between Tumor and Normal Tissues

**Figure 1:**
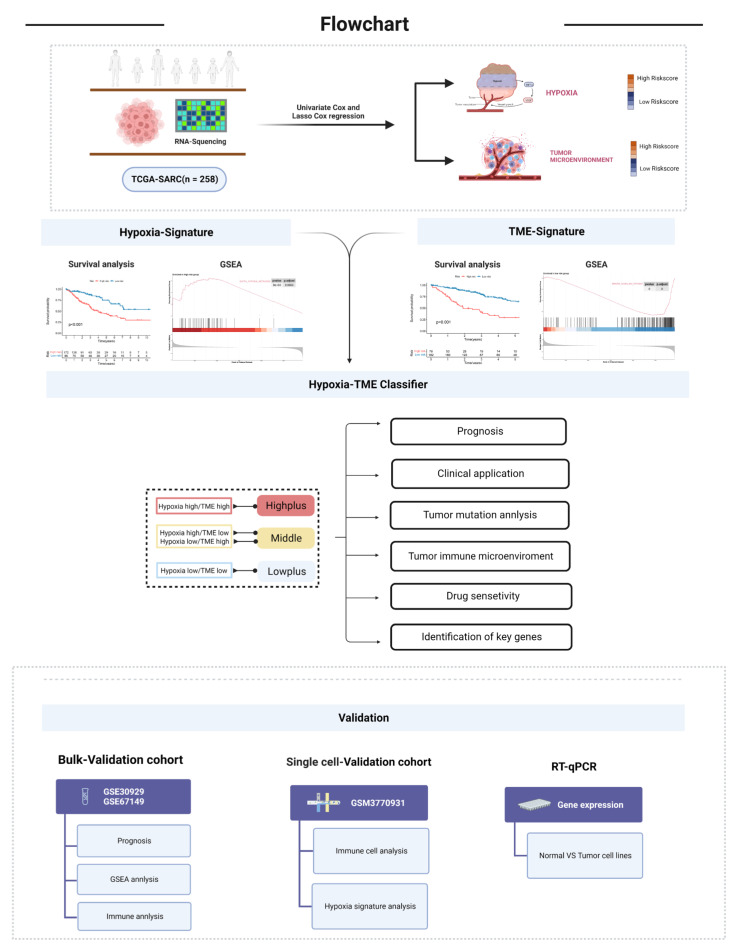
Flow chart of the work process.

**Figure 2:**
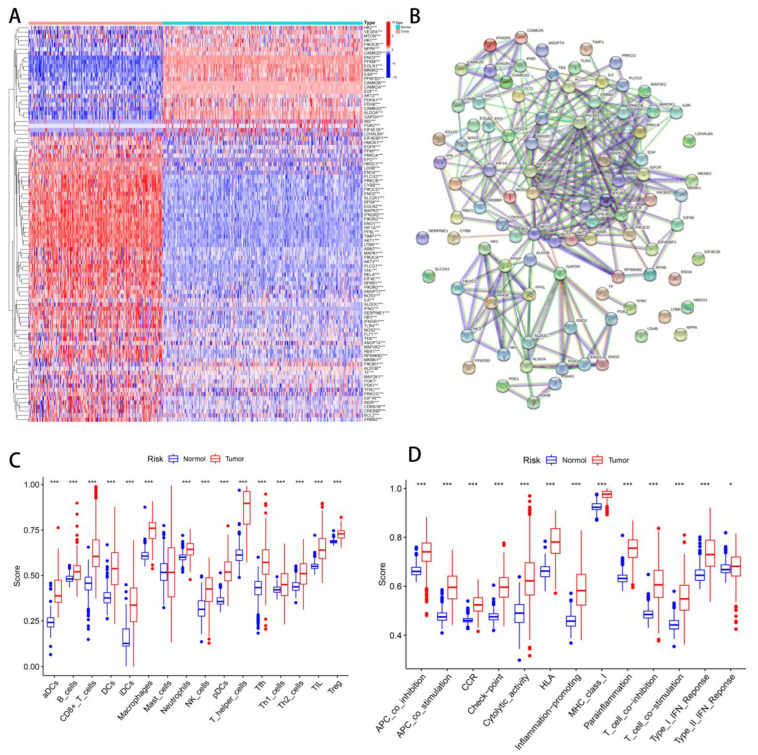
Differentiate hypoxia/TME between normal and tumor tissues. (**A**) Heatmap of hypoxia-related DEGs in normal and tumor tissues. (**B**) PPI network of 94 DEGs. (**C**) Differential analysis of immune cells between normal tissues and tumors. (**D**) Difference analysis of immune function between normal tissues and tumors. ns, *p* ≥ 0.05; *, 0.01 ≤ *p* < 0.05; **, 0.001 ≤ *p* < 0.01; ***, 0.0001 ≤ *p* < 0.001.

An overview of the study is illustrated in Figure 1. A total of 109 hypoxia-related genes were studied across STSs and normal tissues (TCGA-SARC and GTEx, Table S2). A total of 65 genes were upregulated in STS patients, while 28 genes were downregulated (Figure 2A). STRING’s database was used to create the protein-protein interaction (PPI) network (Figure 2B). The analysis was used to integrate 94 differential genes (DEGs) into the PPI network. 

Quantifying immunity cell infiltration and function signatures were achieved using a ssGSEA algorithm. An analysis of 28 immune cell types infiltrating and their functions from 258 SARC patients and 395 non-SARC controls was conducted. The immune cells of normal and tumor tissues differed significantly, except mast cells (Figure 2C). A significant difference was also observed between normal tissue and tumors in regard to all immune functions (Figure 2D). 

### 3.2. Identify the Prognostic Value of Hypoxia and TME 

In order to develop a method that could indicate the status of tumor hypoxic genes and TME cells, 483 patients were studied. The training cohort consisted of 258 patients (TCGA-SARC) while the test cohort consisted of 225 patients (GSE30929 and GSE63157).

Prognostic value analysis was performed on 109 hypoxia-related genes and 64 types of TME cells for patients in the TCGA cohort. An analysis of the effect of 109 hypoxia genes on prognosis was conducted using univariate Cox regression. We strictly screened seven genes for further investigation based on *p* values less than 0.01. As a result of Lasso Cox regression analysis, six hypoxia genes were identified, and a gene signature was constructed related to hypoxia (Figure S1A,B). The hypoxia risk score can be calculated using the following formula: Hypoxia risk score = (0.197549807928693×ENO1) + (−0.206747563778232×IL6R) + (0.0989750992653028×PGK1) + (0.63033060010372×PLCG1) + (−0.243819387058076×PLCG2) + (−0.0215727172904993×PRKCB).

Similarly, 12 TME cells could be identified based on a univariate Cox analysis with a *p*-value of 0.05. As a final step, the cellular features associated with TME were calculated by Lasso analysis with 8 TME cells (Figure S1C,D). The formula for calculating TME risk score: TME risk score = (−0.723923954×Chondrocytes) + (−27.61708633×CMP) + (−0.893613093×HSC) + (−0.068009607×iDC) + (−6.208938889×Macrophages M2) + (4.513782215×Neurons) + (−2.560169253×NKT) + (1.677057993×Th1 cells).

**Figure 3:**
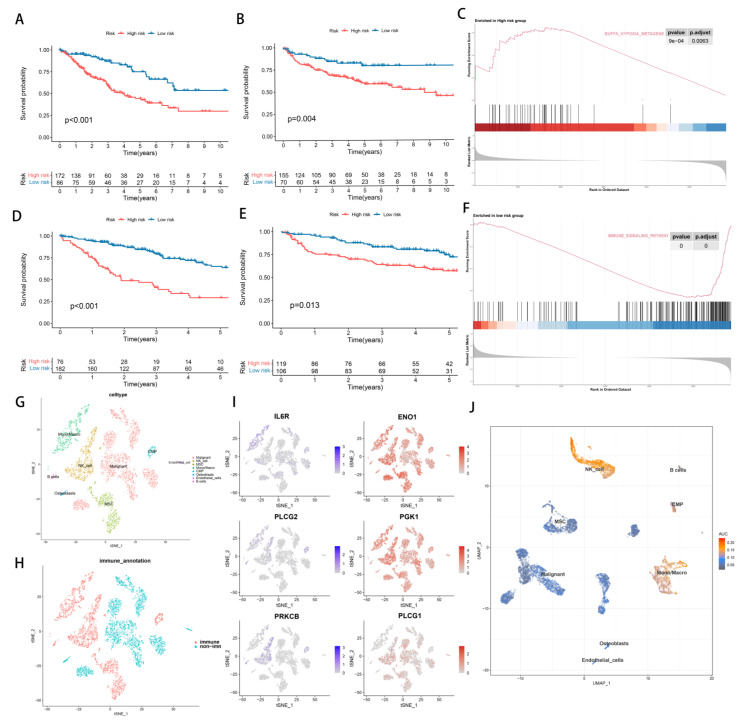
The establishment and identification of hypoxia signatures and TME signatures. (**A**) KM curve of hypoxia−related signature in training cohort. (**B**) KM curve of hypoxia−related signature in testing cohort. (**C**) GSEA enrichment map of DEGs in hypoxia−related subgroups. (**D**) KM curve of TME−related signature in training cohort. (**E**) KM curve of TME−related signature in testing cohort. (**F**) GSEA enrichment map of DEGs in TME-related subgroups. (**G**–**J**) Single cell analysis of GSM3770931 data.

We divided 258 patients with STSs into the low hypoxia risk group (*n* = 86) and the high hypoxia risk group (*n* = 182) according to the best cut-off point of hypoxia score. Similarly, we divided STSs patients into low TME risk group (*n* = 182) and high TME risk group (*n* = 76) (Figure S2A,B). Principal component analysis (PCA) and t-distribution random neighborhood embedding (t-sne) can clearly distinguish between the two groups by the hypoxia classifier, as well as by the TME classifier (Figure S2C). A significant difference in KM plots between high-risk score groups and low-risk score groups was observed in both classifiers of the TCGA-SARC dataset. (*p* < 0.001, Figure 3A,D). Statistics show that low-risk patients have a statistically longer survival time. For verifying the accuracy of the hypoxia classifier and TME classifier, we calculated the risk score of GSE30929 and GSE63157 obtained from the GEO database. PCA and t-SNE also illustrated the optimal degree of discrimination between the two groups (Figure S2D). A significant reduction in survival time was found in the groups with high hypoxia (*p* = 0.004, Figure 3B) and TME risk (*p* = 0.013, Figure 3E). Furthermore, tumors with high hypoxia scores exhibited a significant increase in genes associated with the hypoxia pathway (Figure 3C). Similarly, tumors with low TME risk had a significant increase in immune pathways (Figure 3E). A significant contribution of this study is the analysis of single-cell data obtained from 12 synovial sarcoma patients in order to verify the reliability and necessity of studying hypoxia and TME. We began by analyzing the T-SNE and annotating the clusters (Figure 3G,H and Figure S3C). It was observed that hypoxia-causing genes are widely expressed in a wide variety of cells, while hypoxia-reducing genes are only expressed in immune cells (Figure 3I and Figure S3D). Furthermore, AUCell results demonstrated that immune pathways are enriched where immune cells are located, especially NK_cell (Figure 3J).

### 3.3. Establishment of Hypoxia-TME Classifier

**Figure 4:**
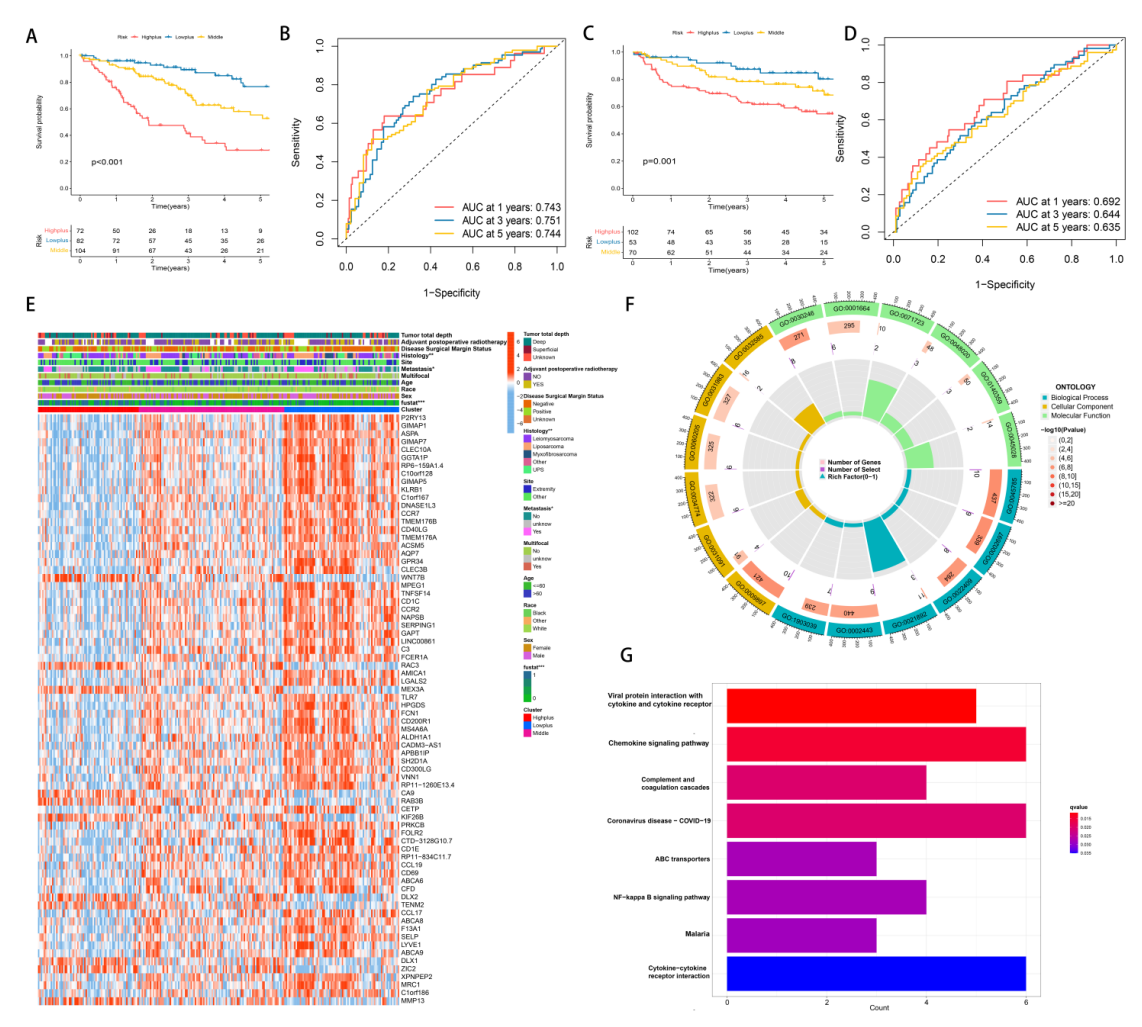
The establishment and identification of Hypoxia-TME classifier. (**A**) KM curve of Hypoxia-TME subgroups in training cohort. (**B**) ROC curve of Hypoxia-TME subgroups in training cohort. (**C**) KM curve of Hypoxia-TME subgroups in testing cohort. (**D**) ROC curve of Hypoxia-TME subgroups in testing cohort. (**E**) Heatmap of clinical characteristics of combined DEGs of Hypoxia-TME-related subgroups. (**F**) GO enrichment analysis circle of DEGs of Hypoxia-TME subgroups. (**G**) KEGG enrichment analysis of DEGs of Hypoxia-TME subgroups. ns, *p* ≥ 0.05; *, 0.01 ≤ *p* < 0.05; **, 0.001 ≤ *p* < 0.01; ***, 0.0001 ≤ *p* < 0.001.

As a result of the above results, we considered whether the hypoxia score and TME score could be combined to further subdivide STS. This is due to the fact that a more precise subgroup classification can facilitate the discovery of mechanisms and the development of effective medications. Around immune cells, immune pathways and genes that reduce hypoxia risk were expressed. Additionally, the hypoxia route was enriched in the hypoxia high-risk group, whereas the immunological pathway was enriched in the TME low-risk group. All of these suggest that combining them will have a greater impact on grouping. Consequently, the Hypoxia-TME classifier combines the Hypoxia score with the TME score, resulting in a three-part classification of patients: Highplus (high hypoxia score/high TME score), Middle (high hypoxia score/low TME score, and low hypoxia score/high TME score) and Lowplus (low hypoxia score/low TME score). Based on Hypoxia-TME classifier results in TCGA-SARC cohorts (*n* = 258), a statistically significant difference was seen in prognoses (*p* < 0.001, Figure 4A). We evaluated the model’s performance using the area under the curve (AUC) of its time-dependent receive operating characteristic (ROC) curve ([AUC]: 1-year = 0.743, 3-year = 0.751, and 5-year = 0.744, Figure 4C). Our validation cohort of GEO was also grouped in the same way to test Hypoxia-TME’s accuracy. Additionally, we calculated the AUC and performed survival analysis. The KM curves among the three groups were significantly different, and the survival time of the Lowplus subgroup was significantly longer (*p* = 0.001, Figure 4B). AUCs were generally satisfactory ([AUC]:1-year [AUC] = 0.692, 3-year = 0.644, 5-year = 0.635, Figure 4D). We added further analysis to the Middle subgroup, and there is no significant difference between the high-hypoxia/low-TME and low-hypoxia/high-TME subgroups (Figure S3A,B). Therefore, the follow-up analysis was based on three subgroups.

We used the limma package to analyze differential transcriptional expression using log2FC > 1 and *p* < 0.05. We compared these three groups on a pairwise basis. Our final selection of differential genes came from the intersection of three sets, totaling 74 DEGs. In this study, DEGs were combined with clinical features and represented by heatmaps (Figure 4E). The Lowplus group showed high expression of most genes, indicating that many of them were protective.

To identify possible hypoxia-TME-related pathways and mechanisms, we further analyzed these 74 DEGs using GO and KEGG enrichment analysis. Based on GO enrichment analysis, we found that DEGs were mainly involved in the positive regulation of cell adhesion (GO:0045785), external side of the plasma membrane (GO:0009897) and carbohydrate binding (GO:0030246) (Figure 4F). In addition, KEGG showed significant enrichment in DEGs in Viral protein interaction with cytokine and cytokine receptor, chemokine signaling pathway, and complement and coagulation cascades (Figure 4G).

### 3.4. Mutation Analysis and Establishment of Hypoxia-TME Prognostic Model

**Figure 5:**
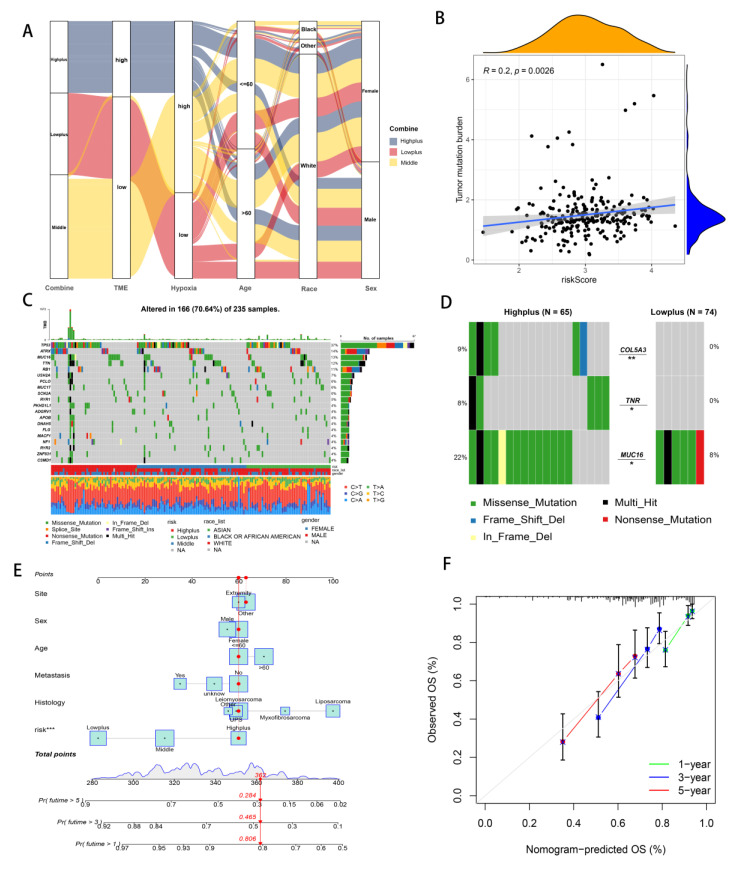
Analyzing Hypoxia-TME information to establish a prognosis model. (**A**) Alluvial diagram illustrating the relationship of Hypoxia/TME-based cluster distribution, different hypoxia and TME risk subgroups and clinical characteristics. (**B**) Relationship between somatic mutation and risk score in training cohort. (**C**) The somatic mutation frequency of three risk groups in TCGA-SARC cohort. (**D**) Significantly different mutant genes between the Highplus and Lowplus groups. (**E**) Nomogram predicting 3-year and 5-year survival rates of STS patients. (**F**) Calibration curve for predicting OS rate of STS patients. ns, *p* ≥ 0.05; *, 0.01 ≤ *p* < 0.05; **, 0.001 ≤ *p* < 0.01; ***, 0.0001 ≤ *p* < 0.001.

An alluvial diagram illustrated the relationships among cluster distributions according to the Hypoxia-TME classifier, hypoxia-related risk category, TME-related risk category, and clinical features (Figure 5A). Hypoxia-TME subgroups were investigated to determine whether they exhibited different tumor somatic alterations. Based on data from the TCGA-SARC cohort, the top 20 variant mutations were identified (Figure 5C). It was found that 166 (70.64%) of 237 samples of sarcoma had mutations and that TP53 was the most commonly mutated gene (37%). In comparison with the other two subgroups, mutations in the Highplus subgroup were significantly higher. Tumor mutation burden were significantly associated with increased Hypoxia-TME-related risk score (R = 0.02, *p* = 0.0026, Figure 5B). We further analyzed the mutations in the Highplus and Lowplus subgroups, in which COL5A3, TNR and MUC16 had significant mutational differences (Figure 5D and Figure S3G).

As part of the evaluation factors, the risk system was included as part of an attempt to further study the potential value of its clinical application. The prognostic model was further enhanced using multivariate Cox regression analysis, which combined clinical features and the Hypoxia-TME risk system. Because the Hypoxia-TME model was established, we intuitively developed this new clear nomogram, which is complementary to the model and has clinical utility. (Figure 5E). Based on nomogram calibration, it has been shown that 3-year and 5-year OS rates are reasonably well predicted (Figure 5F).

### 3.5. Subgroups of Hypoxia-TME Display Distinct Immune Responses

**Figure 6:**
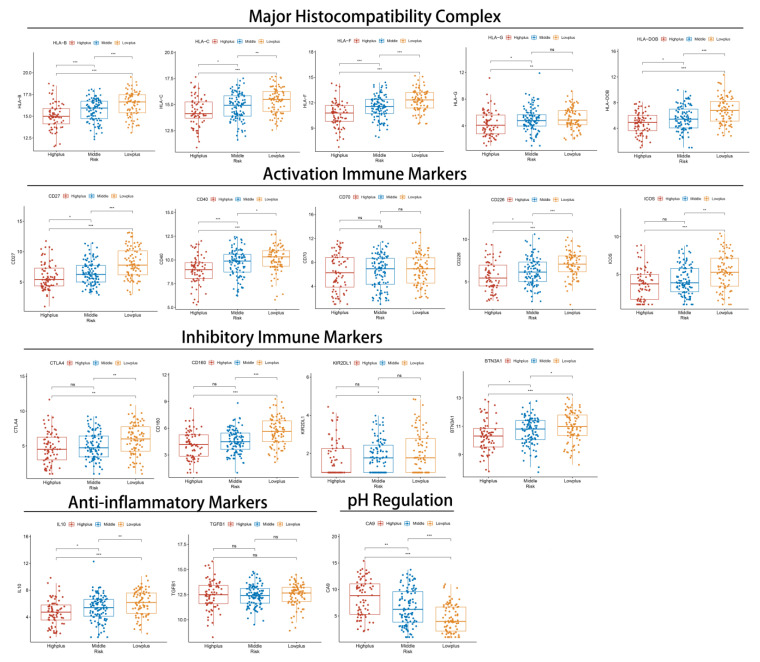
The relationship between immune-related markers and Hypoxia-TME classifiers in three subgroups. Normalized expression of selected markers represented by box and whisker plots. ns, *p* ≥ 0.05; *, 0.01 ≤ *p* < 0.05; **, 0.001 ≤ *p* < 0.01; ***, 0.0001 ≤ *p* < 0.001.

Further investigation of immune response genes in different subgroups was then conducted from several perspectives: major histocompatibility complex (MHC), inhibitory immune markers (IIM), activation immune markers (AIM), anti-inflammatory markers, and pH regulation marker (Figure 6 and Figure S3E,F). In the Highplus subgroup, we observed significant decreases in the expression of all MHC, most AIM (except CD70), and all IIM compared with the Lowplus subgroup. It was also noteworthy that there was a significant gradient between the three subgroups in most of these genes (HLA-B, HLA-C, HLA-F, HLC-DOB, CD27, CD40, CD226, and BTN3A1). All three subgroups expressed significantly different levels of IL-10 (anti-inflammatory gene). The Highplus subgroup demonstrated a lower expression of IL-10 than the other two subgroups. Additionally, CA9 expression was found to be significantly higher in the Highplus subgroup, which regulates pH.

**Figure 7:**
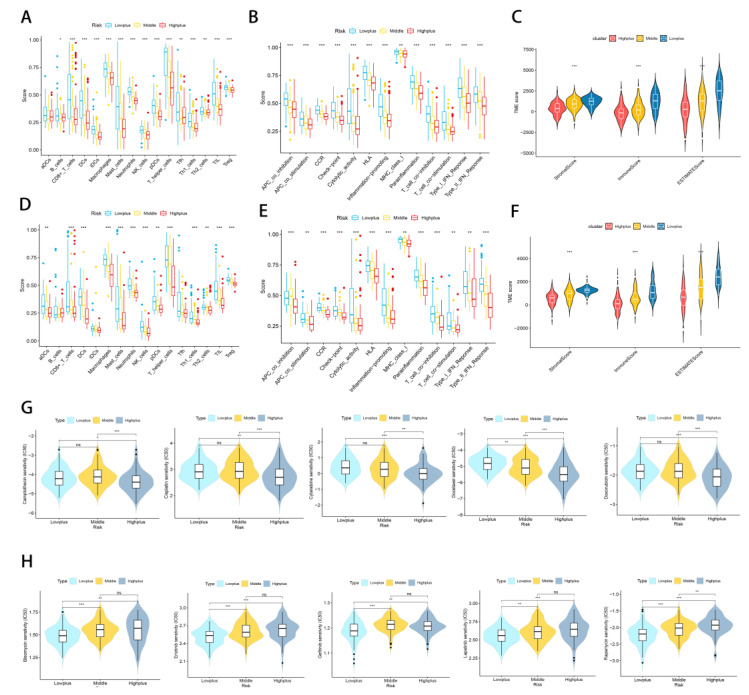
Subgroups of Hypoxia-TME display distinct immune responses and immune/chemotherapy sensitivity. (**A**,**D**) Comparative analysis of immune cells and immune functions in three different training risk groups. (**B**,**E**) Comparative analysis of immune cells and immune functions in three different testing risk groups (**C**) Analyze the immune status of each subgroup in the training set using ESTIMATE. (**F**) Analyze the immune status of each subgroup in the testing set using ESTIMATE. (**G**) Drugs that are highly effective for the Highplus group. (**H**) Drugs that are highly effective for the Lowplus group. ns, *p* ≥ 0.05; *, 0.01 ≤ *p* < 0.05; **, 0.001 ≤ *p* < 0.01; ***, 0.0001 ≤ *p* < 0.001.

Aside from analyzing immune response genes, we also examined immune cells and functions. By analyzing immune infiltration and function by ssGSEA, we compared the immune activity of three subgroups. The results indicated that there were significant differences between the three subgroups in most immune cells (except aDCs) and immune functions of the TCGA-SARC cohort (Figure 7A,B). An analysis of the test cohort dataset (*n* = 225) was also conducted to determine immune activity. Consistent with the results of the training cohort (Figure 7D,E). Moreover, we looked at how immune cells are related to risk scores (Figure S4). Whether in the train set or the test set, as a result of the validation of immune status using ESTIMATE, ImmuneScore, StromalScore, and ESTIMATEScore were significantly lower in the Highplus subgroup samples as compared to the Lowplus subgroup samples (Figure 7C,F)

### 3.6. Treatment Response Prediction with Hypoxia-TME

Considering the excellent grouping results above, it is important to determine treatment methods for each subgroup. In view of the fact that immune/chemotherapy is a common treatment for STS, we evaluated the response of three Hypoxia-TME-related risk subtypes to drugs. Based on a 10-fold cross-validation analysis of the GDSC cell line dataset, we trained a predictive model using ridge regression with satisfactory predictive accuracy. According to our results, traditional chemotherapy drugs generally have good efficacy in the Highplus subgroup, while immunotherapy has a greater possibility in the Lowplus subgroup. A total of 10 drugs were screened for the Highplus and Lowplus subgroups (Figure 7G,H). The Highplus subgroup was effectively treated with five of these drugs (Figure 7G, *p* < 0.05, Camptothecin, Cisplatin, Cytarabine, Docetaxel, and Doxorubicin). Subgroup Lowplus responded well to five other drugs (Figure 7H, *p* < 0.05, Bleomycin, Erlotinib, Gefitinib, Lapatinib, and Rapamycin).

### 3.7. Differences in Gene Expression within Subgroups and the Identification of Key Genes

**Figure 8:**
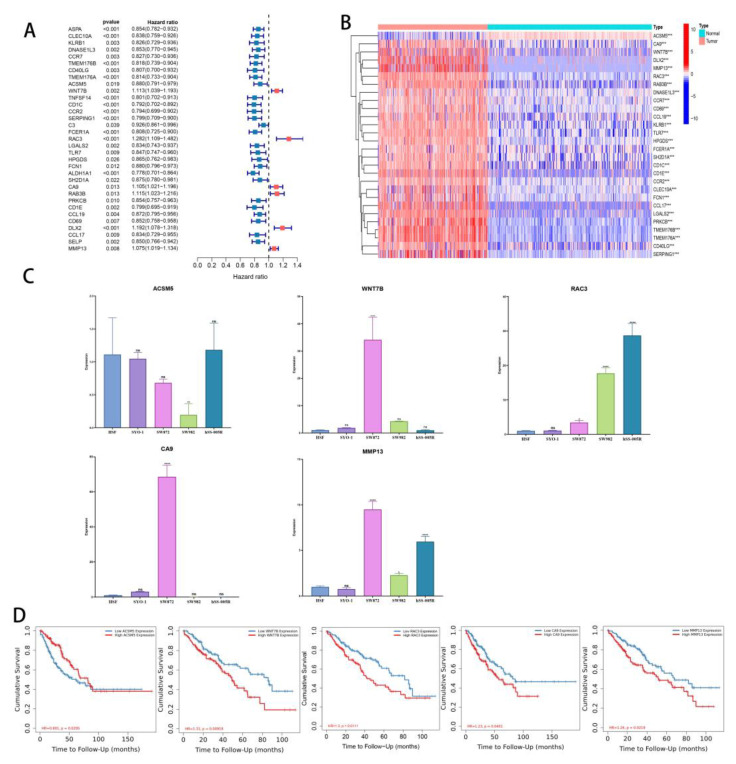
Identification of key DEGs. (**A**) Forest map of prognostic-related DEGs. (**B**) Heatmap of differential gene expression related to prognosis in normal tissues and tumors (**C**) Validation of ACSM5, WNT7B, RAC3, CA9, and MMP13 expression in cell lines by using RT-qPCR. (**D**) KM curves of TCGA-SARC patients divided by ACSM5, WNT7B, RAC3, CA9, and MMP13. ns, *p* ≥ 0.05; *, 0.01 ≤ *p* < 0.05; **, 0.001 ≤ *p* < 0.01; ***, 0.0001 ≤ *p* < 0.001; **** *p* < 0.0001.

The previous analysis identified 74 DEGs in the three subgroups. Based on a prognostic analysis using a 0.05 *p*-value, 33 DEGs with prognostic significance were identified (Figure 8A). A significant difference was found between tumor and normal tissue expression levels of these 33 genes (Figure 8B). The WNT7B, RAC3, CA9, RAB3B, DLX2, and MMP13 genes all had a significantly increased expression in tumors and were significantly associated with poor prognoses. In addition, a significant overexpression of ACSM5 was observed in tumors, and it was protective of prognosis.

Quantitative RT-PCR was used to analyze these genes, and ACSM5, WNT7B, RAC3, CA9, and MMP13 were all consistent with the data (Figure 8C). According to our pan-cancer analysis, these five genes differ significantly in most tumors (Figure S5A–E). Finally, we tested the layering effect of the five genes using TIMER2.0. Segmentation points were based on the median gene expression. Each gene stratification resulted in significant differences (Figure 8D).

## 4. Discussion

Researchers are gaining a deeper understanding of hypoxia and TME in relation to the prognosis and treatment of cancer patients due to an explosion of research [18,43]. There are significant differences between normal tissues and tumors in hypoxia genes and TME cells, according to our results. Despite this, few studies had integrated hypoxia and TME signatures for predicting prognosis and treatment outcomes. Using the Hypoxia-TME classifier, we systematically assessed the prognostic and therapeutic value of hypoxic TME in large STS cohorts. 

The first step was to establish the hypoxia signatures and TME signatures. For hypoxia signature, a protective role for prognosis is played by the hypoxia genes IL6R, PLCG2, and PRKCB. In single cell analysis, these three genes showed a high expression only in immune cells, supporting the hypothesis that the infiltration of immune cells benefited the patient’s prognosis. PRKCB is a regulator of angiogenesis in the hypoxia signature genes [44], which demonstrates the important role of angiogenesis in tumorigenesis development. The strong correlation between the regulation of angiogenesis-related factors and immune cells also improves the strong evidence for the subsequent analysis of TME. However, many angiogenesis-related genes have not been included in the hypoxic signature, and it is worthwhile to investigate the relationship between these genes and TME as one of the future research directions. A favorable role was played by Chondrocytes, CMP, HSC, iDC, Macrophages M2 and NKT cells among the eight TME cell types in the STS cohorts studied. In addition to lymphocytes and myeloid cells, nonimmune cells within tumors were also considered prognostic, providing further support for our hypothesis that nonimmune cells also influence cancer prognosis. For external verification, we compared the performance of the two classifiers using fusion data from GSE63157 and GSE30929. Furthermore, GSEA results revealed significant enriched hypoxia-related pathways in the high hypoxia risk group, and enriched immune response-related pathways in the low TME risk group. 

We then constructed a classifier using Hypoxia/TME, which produces good results both in training and testing. In three subgroups, 74 DEGs were identified, with the majority expressed at a high level in the Highplus subgroup. The mutational status of subgroups was also studied. According to our analysis, the gene mutations in the Highplus subgroup were significantly higher, and their correlation with the risk score proved our classification system to be superior. COL5A3, TNR and MUC16 have significant mutational differences between the Highplus and Lowplus subgroups. These three genes encode proteins that are inextricably linked to the extracellular matrix; COL5A3 is involved in encoding a fibrillar collagen molecule [45], TNR encodes a member of the tenascin family of extracellular matrix glycoproteins [46], and MUC16 encodes a protein belonging to the mucin family [47]. Meanwhile, the extracellular matrix is an acidic hypoxic environment. The results suggest that the causal link between gene mutations and hypoxia in the sarcoma can be explained by the intrinsic mechanism of these three mutated genes. Additionally, using a combination of hypoxia/TME risk grouping, we have developed a clinical prediction model. The significance of risk grouping as a prognostic factor demonstrated that we could apply our grouping in the clinic.

A further finding revealed that both activating and inhibitory immune markers were exceptionally high in the Lowplus group. Based on the result, an improved antitumor immune response is likely to be restored in Lowplus patients through immune checkpoint blockade. Consequently, the Hypoxia-TME classifier might be applied to cancer patients stratified before immunotherapy. A statistically higher CA9 expression in the Highplus subgroup can also be attributed to an acidic extracellular milieu, which might contribute to poor tumor differentiation and development as well as increased tumor growth [48,49]. A further demonstration of the classifier’s prediction ability was provided. We also evaluated the immune infiltration status in the training cohort and the testing cohort. There is a significant increase in immune cell infiltration and immune score in the Lowplus group, which indicates that our classifier has a comprehensive discrimination ability. To determine which subgroups are more responsive to treatment, we evaluated the responses of the three subgroups. The Lowplus group exhibited greater efficacy with drugs such as Bleomycin, Erlotinib, Gefitinib, Lapatinib, and Rapamycin, while the Highplus group displayed greater efficacy with drugs such as Camptothecin, Cisplatin, Cytarabine, Docetaxel, and Doxorubicin. The main reasons for different drug sensitivity of tumors include the genomic drivers, immune system and TME [50]. Our grouping was based on hypoxia and TME, and there were significant differences in gene mutation among subgroups. Birkbak NJ et al. found that mutations in the BRCA gene resulted in defective DNA repair and thus predicted susceptibility to DNA damaging agents; and they also found that accumulation of allelic imbalance was a marker of platinum sensitivity marker [51]. Our results suggest that the higher sensitivity to DNA-damaging drugs in the high mutation group (Highplus) may also be due to mutations in certain genes, and the three genes (COL5A3, TNR and MUC16) we identified may be related to potential mechanisms of drug sensitivity. As a result of our grouping, patients’ prognoses can be evaluated, and their precise treatment can also be facilitated.

In order to further analyze the mechanism, we searched for the key genes that differed between the three subgroups. Based on 74 DEGs, 33 genes were screened for prognostic potential. A significant difference was found between the expression of 33 prognosis-related genes in normal tissues and tumors. Seven genes showed expression differences that were consistent with the prognosis. In our RT-qPCR analysis, ACSM5, WNT7B, CA9, MMP13, and RAC3 showed a consistent trend, indicating that our classification accuracy was high. Based on the median expression of these five genes, we found that the prognosis results were significantly different, which further explained the importance of these five genes in the three subgroup differences. Studies have shown that WNT7B, CA9, MMP13, and RAC3 are associated with poor outcomes in sarcomas. In human OS, Wnt inhibitory factor 1 (WIF1) is epigenetically silenced while Wnt target genes are amplified [52,53,54]. Molecular pathways downstream of Wnt ligands play a critical role in tumorigenesis and are evolutionarily conserved, and Wnt signaling and Loxl2 promote aggressive osteosarcoma [55]. It has been suggested that CA9 may be used as an intrinsic marker of hypoxia in patients with deep, large, and high-grade STS, which may lead to a poor prognosis [56]. However, it needs to be determined whether CA9 is an independent prognostic factor in STS by larger studies. Several studies have shown that osteosarcomas with high MMP13 expression have poor outcomes [57,58]. Recently, researchers found that mutations that activate RAC GTPases were therefore detected at a low frequency in a variety of human cancers [59]. Even though ACSM5 is rarely studied in STS, it also points the way forward. Four other cancer-promoting factors may be potential therapeutic targets and research genes in the future.

In our study, we acknowledge that there are some limitations. As an initial step, we need to confirm Hypoxia-TME signatures in tumor samples (biopsies) using immunofluorescence or flow cytometry. The second step is to perform an in-house cohort evaluation to further test the performance of the classifier due to the limitations of public datasets. 

## 5. Conclusions

To summarize, displaying the hypoxia and cellular signatures within tumor microenvironments can help to predict prognoses. The classifier also identifies the sensitivity of drugs in STS, so patients will avoid unnecessary side effects associated with medication. The established nomogram can also improve clinicians’ ability to accurately predict STS patients’ fate, thereby offering them clinical strategies. Furthermore, we have identified key genes that will be very useful in exploring future mechanisms and researching future directions. In order to uncover the pathways in which these genes function, further research and validation are required.

## Figures and Tables

**Table 1:** Sequences of the primers used in RT-qPCR.

Gene	Sequence of Primer
GAPDH	F ^1^: CAGGAGGCATTGCTGATGAT
	R ^2^: GAAGGCTGGGGCTCATTT
ACSM5	F: GGACAGGGACTGTGATGATTCC
	R: CCCTTGGAGCTAGGGAGTCA
WNT7B	F: GAAGCAGGGCTACTACAACCA
	R: CGGCCTCATTGTTATGCAGGT
RAC3	F: TCCCCACCGTTTTTGACAACT
	R: GCACGAACATTCTCGAAGGAG
RA9	F: TTTGCCAGAGTTGACGAGGC
	R: GCTCATAGGCACTGTTTTCTTCC
MMP13	F: ACTGAGAGGCTCCGAGAAATG
	R: GAACCCCGCATCTTGGCTT

^1^ F: Forward primer; ^2^ R: Reversed primer.

## Data Availability

Not applicable.

## Supplementary Materials

The following are available online at https://www.mdpi.com/article/10.3390/cancers14225675/s1, Figure S1. (A) LASSO regression analysis of hypoxia-related genes. (B) Cross-validation method to select optimal genes. (C) LASSO regression analysis of TME cells. (D) Cross-validation method to select optimal TME cells. Figure S2. (A,B) The establishment and identification of hypoxia signatures. (C,D) The establishment and identification of TME signatures. Figure S3. (A) KM curve of high-hypoxia /low-TME and low-hypoxia/high-TME subgroups in training cohort. (B) KM curve of high-hypoxia /low-TME and low-hypoxia/high-TME subgroups in testing cohort. (C) Identification of marker genes in single cells. (D) Expression of hypoxia signature genes in single cells. (E,F) Relationship between risk score and immune checkpoint. (G) Mutations in COL5A3, TNR and MUC16 in Highplus and Lowplus subgroups. Figure S4. (A–N) Assess the relationship between risk score and immune cells. Figure S5. (A–E) Expression analysis of ACSM5, WNT7B, RAC3, CA9 and MMP13 in Pan-cancer. Table S1. RNA sequence data of TCGA-SARC cohort. Table S2. Clinical data of TCGA-SARC cohort. Table S3. RNA sequence data of normal soft tissues. Table S4. RNA sequence data of GSE63157 and GSE30929. Table S5. Survival data from GSE63157 and GSE30929. Table S6. The single-cell dataset of GSM3770931. Table S7. 109 hypoxia-related genes. Table S8. 64 types of TME cells.
