# Supplementary figures and images for: Integrated Analysis of TME and Hypoxia Identifies a Classifier to Predict Prognosis and Therapeutic Biomarkers in Soft Tissue Sarcomas
Source: Cancers (Basel). 2022 Nov 18;14(22):5675. doi: 10.3390/cancers14225675 (PMC9688460; doi:10.3390/cancers14225675)

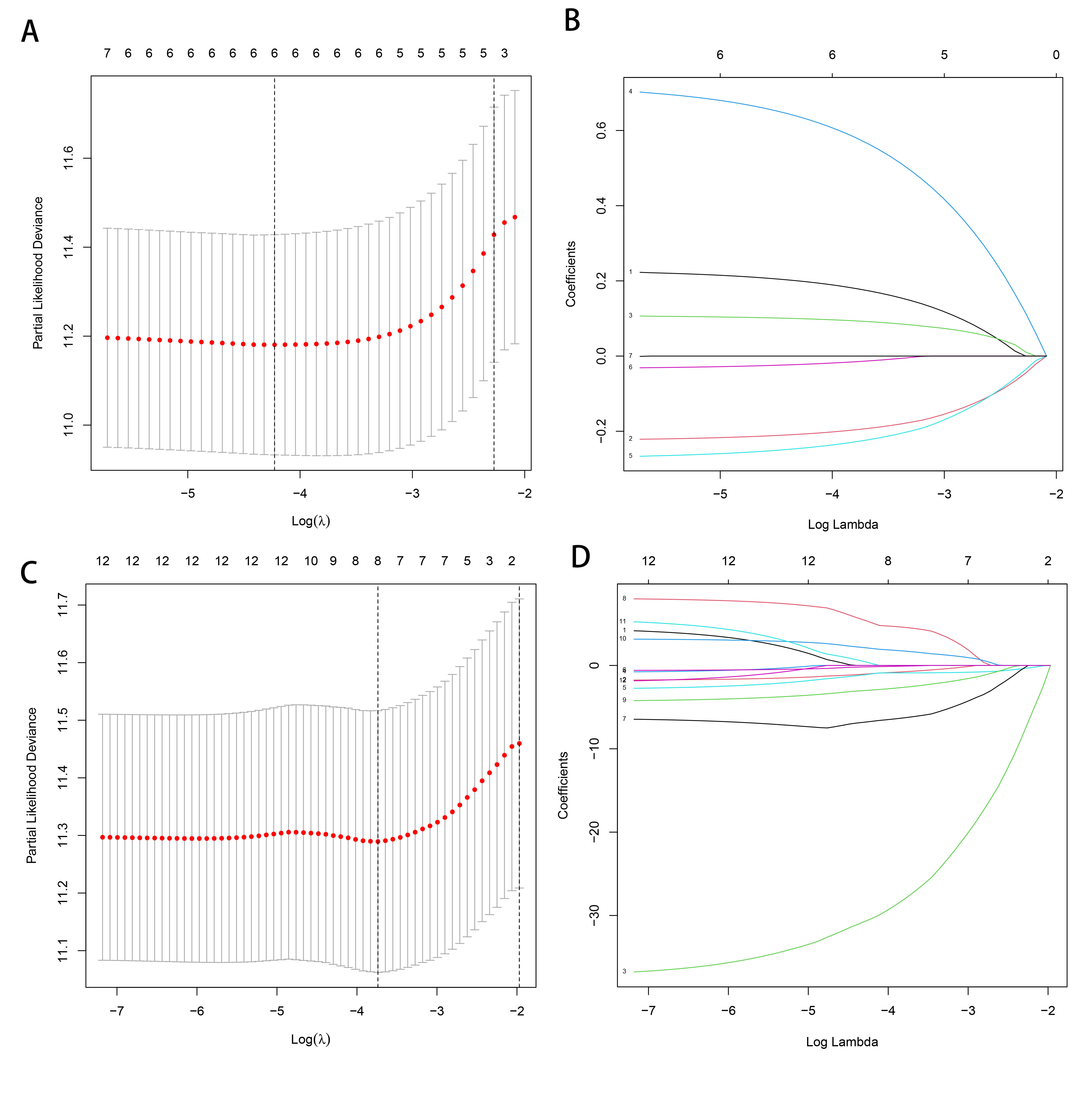

Supplement: Supplementary file 1 [file cancers-14-05675-s001.zip › Supplementary materials/Figure S1.jpg]

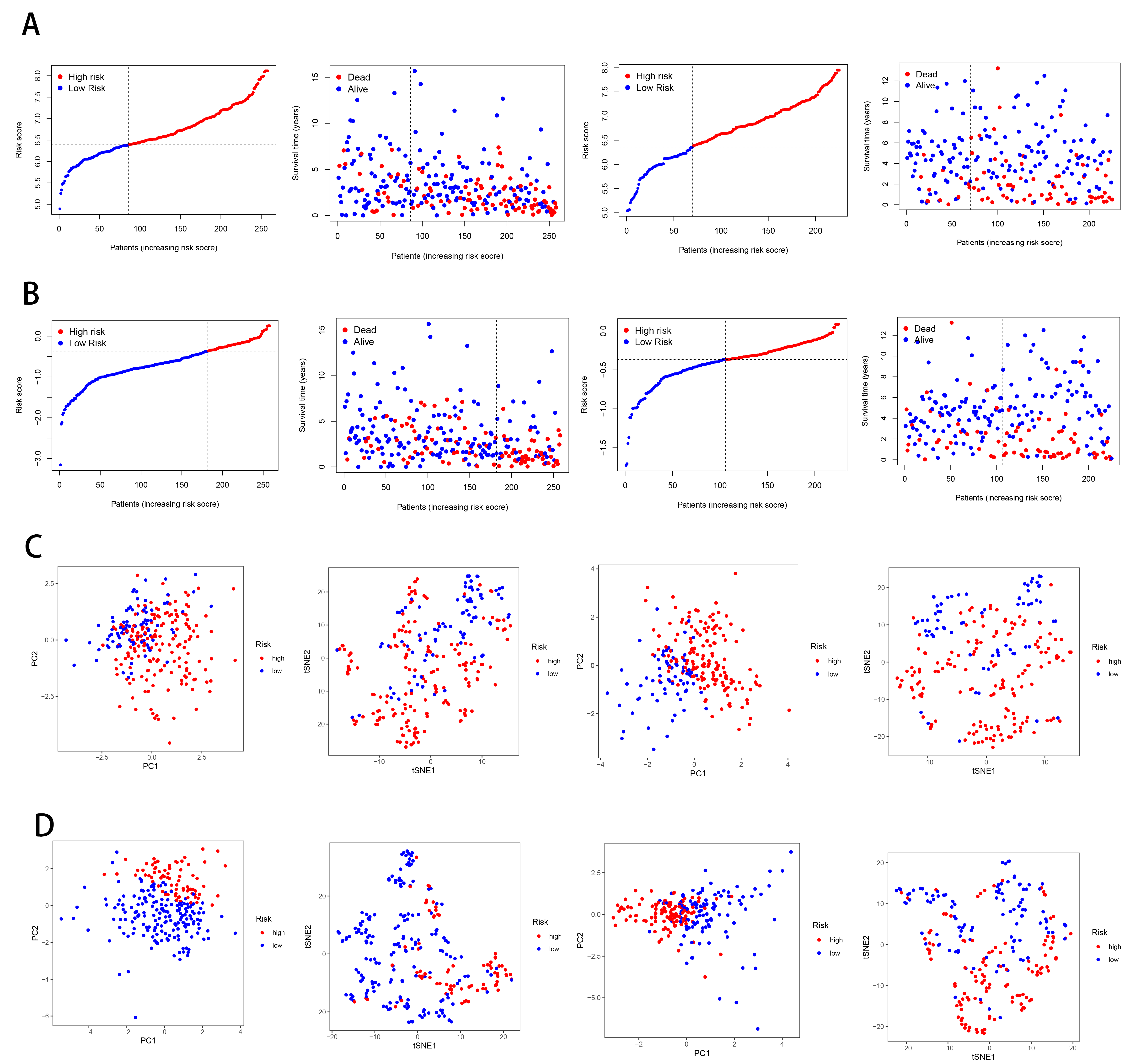

Supplement: Supplementary file 1 [file cancers-14-05675-s001.zip › Supplementary materials/Figure S2.jpg]

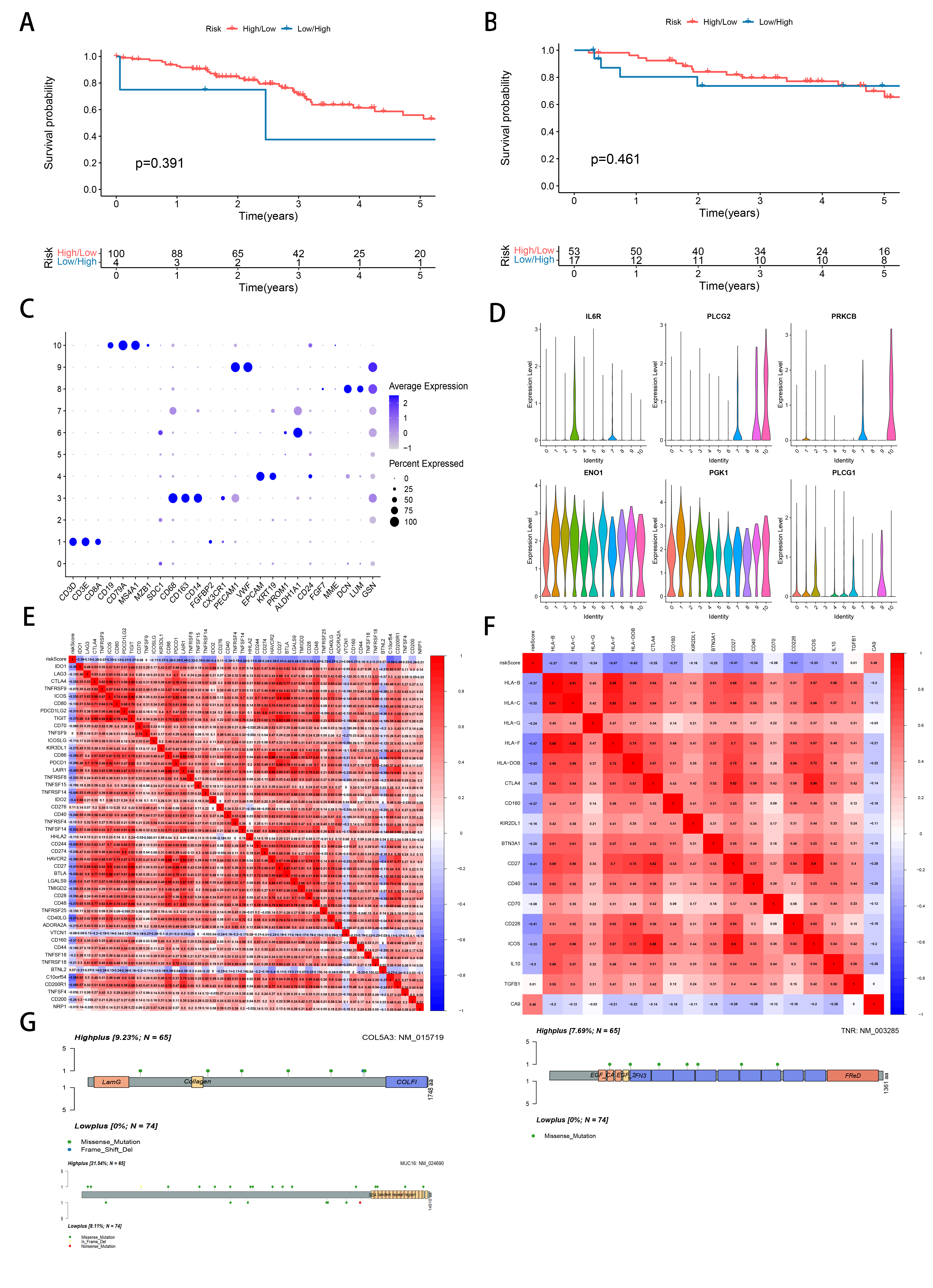

Supplement: Supplementary file 1 [file cancers-14-05675-s001.zip › Supplementary materials/Figure S3.jpg]

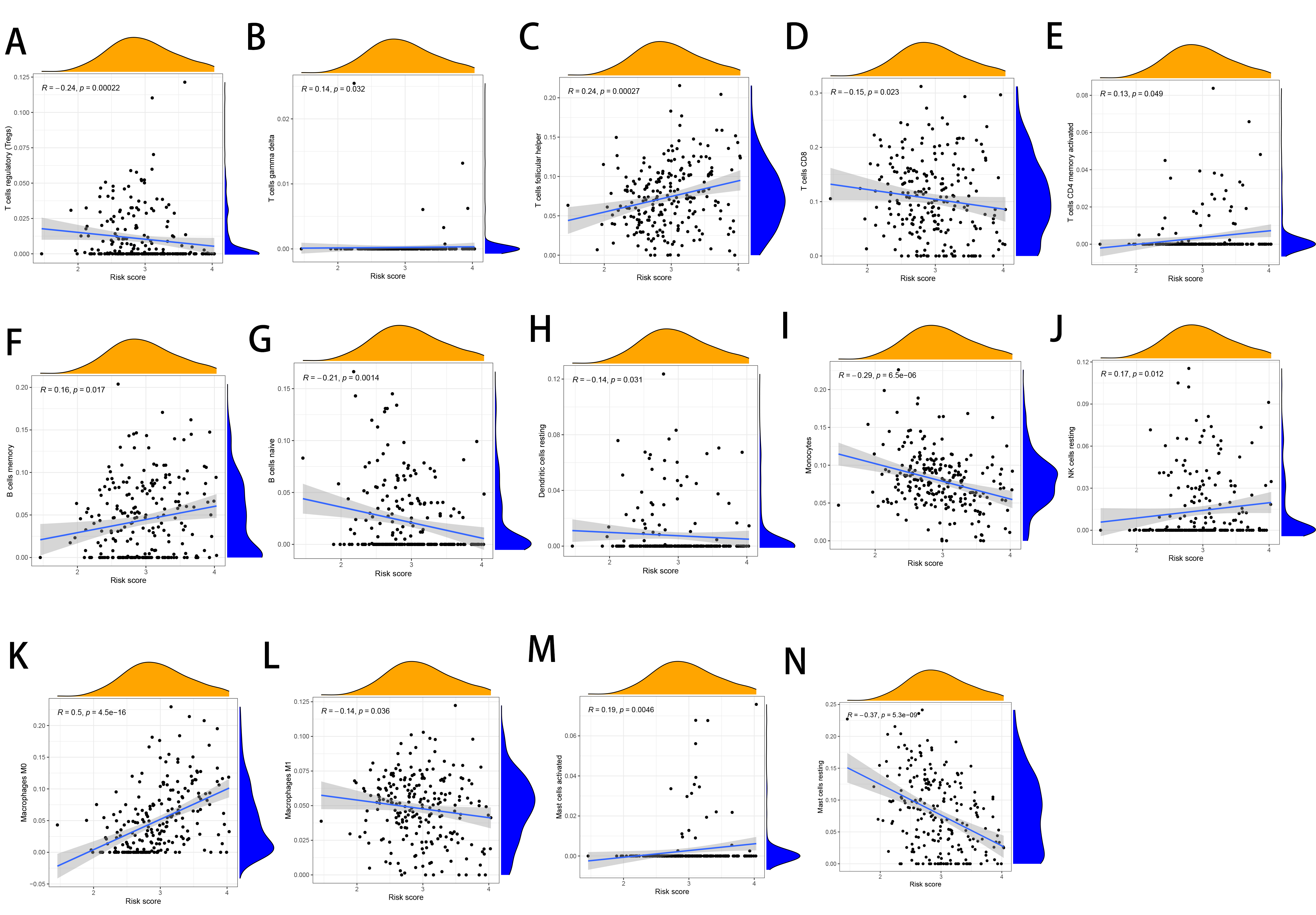

Supplement: Supplementary file 1 [file cancers-14-05675-s001.zip › Supplementary materials/Figure S4.jpg]

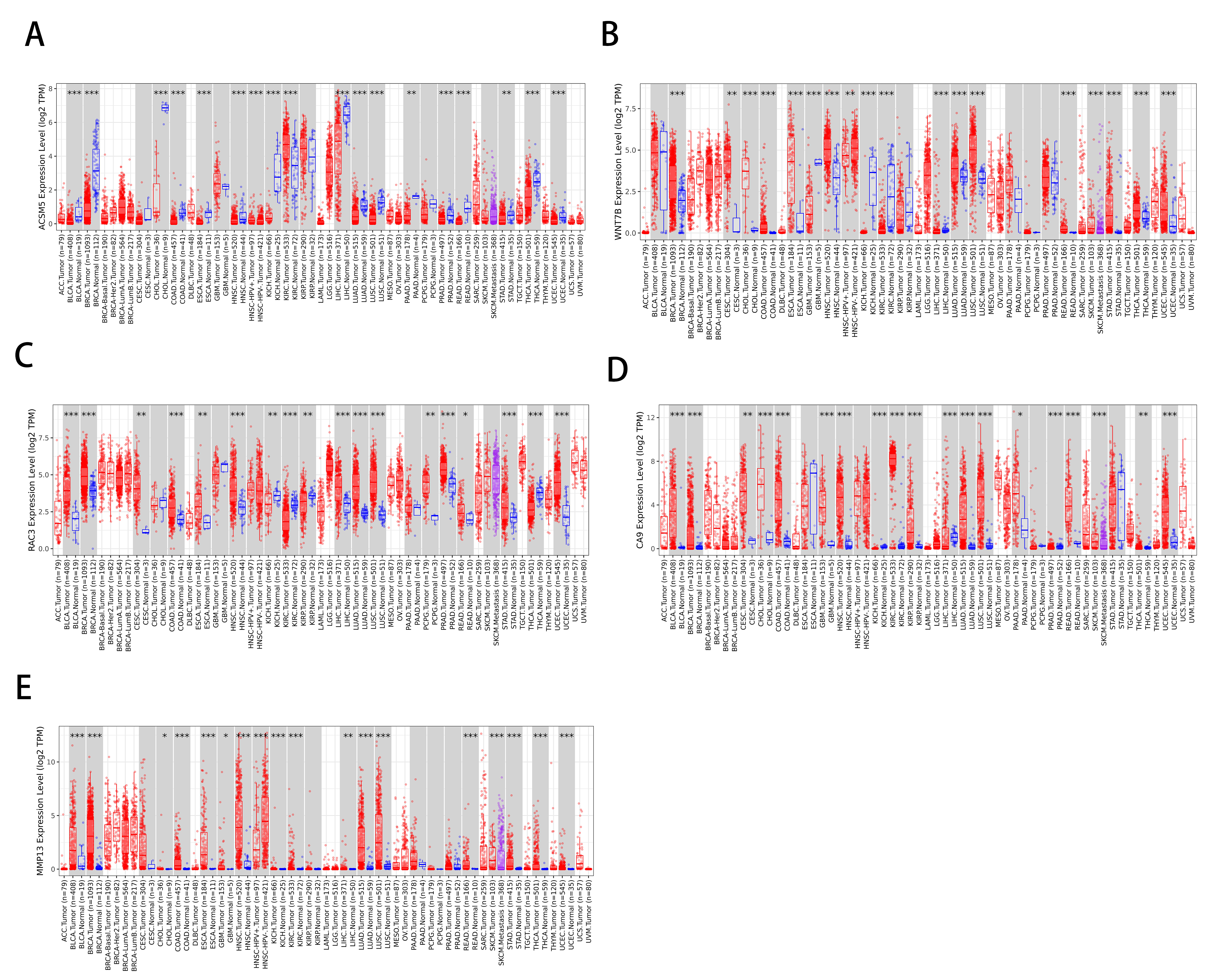

Supplement: Supplementary file 1 [file cancers-14-05675-s001.zip › Supplementary materials/Figure S5.jpg]
